# Hydroxyl Dicarboxylic Acids at a Mountainous Site in Hong Kong: Formation Mechanisms and Implications for Particle Growth

**DOI:** 10.1021/acsenvironau.4c00119

**Published:** 2025-03-14

**Authors:** Hongyong Li, Xiaopu Lyu, Likun Xue, Yunxi Huo, Tianshu Chen, Dawen Yao, Haoxian Lu, Beining Zhou, Hai Guo

**Affiliations:** † Environment Research Institute, 12589 Shandong University, Qingdao 266237, China; ‡ Department of Geography, Faculty of Arts and Social Sciences, 26679 Hong Kong Baptist University, Hong Kong 999077, China; § Department of Civil and Environmental Engineering, 26680 The Hong Kong Polytechnic University,Hong Kong 999077, China; ∥ School of Intelligent Systems Engineering, 26469 Sun Yat-Sen University, Shenzhen 518100, China; ⊥ Southern Marine Science and Engineering Guangdong Laboratory (Zhuhai), Zhuhai 519000, China

**Keywords:** hydroxyl dicarboxylic acid, malic acid, secondary organic aerosol, formation mechanism, machine learning

## Abstract

Secondary organic aerosol (SOA) has been shown to significantly impact climate, air quality, and human health. Hydroxyl dicarboxylic acids (OHDCA) are generally of secondary origin and ubiquitous in the atmosphere, with high concentrations in South China. This study explored the formation of representative OHDCA species based on time-resolved measurements and explainable machine learning. Malic acid, the most commonly studied OHDCA, had higher concentrations in the noncontinental air (63.7 ± 33.3 ng m^–3^) than in the continental air (7.5 ± 1.4 ng m^–3^). Machine learning quantitatively revealed the high relative importance of aromatics and monoterpenes SOA, as well as aqueous processes, in the noncontinental air, due to either shared precursors or similar formation pathways. Isoprene SOA, particle surface area, and ozone corrected for titration loss (O_*x*_) also elevated the concentrations of malic acid in the continental air. Aqueous photochemical formation of malic acid was confirmed given the synergy between LWC, temperature, and O_*x*_. Moreover, the OHDCA-like SOA might have facilitated a relatively rare particle growth from early afternoon to midnight in the case with the highest malic acid concentrations. This study enhances our understanding of the formation of OHDCA and its climate impacts.

## Graphical abstract

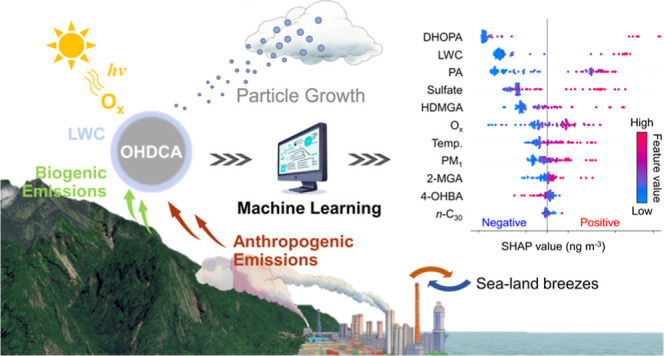

## 1 Introduction

Secondary organic aerosol (SOA) represents a substantial fraction of atmospheric fine particulate matter, with profound implications for climate, air quality, and human health.^1–3^ Hydroxyl dicarboxylic acids (OHDCA) in the atmosphere, such as malic acid and tartaric acid, were observed with high concentrations in some regions and were identified as secondary in origin.^4–6^ Owing to their polar functional groups, including hydroxyl and carboxyl, OHDCA species may significantly contribute to the hygroscopic properties of aerosols. Due to their high polarity and low vapor pressure, dicarboxylic acids can influence the ability of aerosols to act as cloud condensation nuclei (CCN), thereby impacting the climate.^7,8^ Similar effects are also expected for OHDCA with higher proportions of polar functional groups. Additionally, some OHDCA species (e.g., malic acid and tartaric acid) have been found to play a crucial role in the formation and growth of new particles in the atmosphere.^9,10^

These significant effects necessitate the understanding of sources and formation mechanisms of OHDCA. Although they are prevalent in fruits and used as food additives, evidence for their direct release into the atmosphere is lacking.^11^ At a regional background site in South China, OHDCA exhibited the same diurnal patterns (e.g., peaks in the afternoon) as O_*x*_, the sum of ozone (O_3_) and nitrogen dioxide (NO_2_), implying a photochemical source of OHDCA.^12^ Chamber experiments confirmed the formation of malic acid from the photooxidation of toluene, 1,3,5-trimethylbenzene, α-pinene, and isoprene.^13–15^ Besides, OHDCA was generated in ozonolysis experiments involving cycloalkanes and α-pinene.^16^ It was found that the yields of OHDCA from gas-phase oxidations are generally low.^13,16,17^ The detection of malic acid from aqueous-phase photooxidation of glycolaldehyde, methylglyoxal, and glyoxal indicated aqueous processes as an alternative pathway of OHDCA formation.^18–20^ Succinic acid, a highly water-soluble precursor of malic acid, underwent further aqueous-phase oxidation at a rate that surpassed gas-phase processes.^18^ Studies also indicated that the photochemical and aqueous processes in the formation of some SOA species, including OHDCA, might not be separate.^21,22^

In Hong Kong (HK), high levels of OHDCA were observed in different times and spaces using different analytical techniques.^4,12,23^ Using thermal desorption aerosol gas chromatography coupled with time-of-flight mass spectrometry (TAG), we conducted time-resolved measurements of organic aerosol (OA) molecular markers, including OHDCA, at a coastal background site. The results suggested that anthropogenic emissions were likely a primary source of OHDCA precursors and that certain environmental conditions (e.g., high relative humidity, highly aged continental outflows and ship emissions) might have promoted the formation of OHDCA.^12^ A subsequent study at the same site confirmed that the elevated levels of OHDCA were related to aqueous-phase photooxidation of both biogenic and anthropogenic precursors, and the limiting factors for OHDCA formation differed between coastal and continental air.^22^ In summary, the OHDCA are a type of relatively aged SOA species with diverse precursors and complex formation mechanisms. The previous research findings on the chemistry of OHDCA in HK were qualitative in nature, and there is a lack of quantitative understanding of the factors regulating OHDCA concentrations. Moreover, the implications of OHDCA on climate have not been well understood.

In this study, we conducted an intensive field campaign at a mountainous site in HK in November 2021, with time-resolved measurements of submicron particulate matter (PM_1_) components, molecular OA markers, including four OHDCA species, size-resolved particle number concentrations, and other supporting data. The potential precursors and influencing factors of OHDCA formation were identified, and their relative importance and synergistic effects were quantitatively assessed by explainable machine learning. The role of OHDCA-like SOA in particle growth was unraveled in a case with the highest levels of OHDCA for the entire sampling period. The results of this study enhance our understanding of OHDCA formation mechanisms and provide some insights for refining air quality and climate models.

## 2 Methods

### 2.1 Sampling Campaign

With the elevation of 957 m a.s.l., Mount Tai Mo Shan (Mt. TMS) is the highest peak in HK and located in the central part of the land. During November 6–14, 2021, the sampling campaign was conducted at a youth hostel on the mountainside (22.405° N, 114.118° E, 640 m a.s.l.), as shown in Figure S1. To the south of the site, a bustling town with a dense population, advanced transportation networks, and a busy freight harbor lies at the foothills. The mesoscale circulation, i.e., mountain-valley breeze, was shown to be capable of transporting air pollutants from the foot of the mountain to the site.^24,25^ Biogenic emissions were also likely to be a significant source of volatile organic compounds (VOCs) in the study area due to the dense vegetation cover. Affected by the COVID-19, there were few visitors to the youth hostel, except for a small influx of campers during the weekends. Additionally, the site was influenced by inland air from the neighboring mainland to the north, especially in the cool seasons.

The Hybrid Single-Particle Lagrangian Integrated Trajectory (HYSPLIT) model was employed to compute the 48 h backward trajectories of air masses arriving at the site hourly. Meteorological data were sourced from the Global Data Assimilation System archive, with a horizontal resolution of 1° × 1°. To avoid incorrect grid sampling in mountainous and valley areas, atmospheric pressure was used as a proxy for modeling height, as adopted by Lyu et al.^26^ During the sampling period, the average observed atmospheric pressure at the site was 950 hPa. The hourly trajectories were classified into three clusters, according to their origins and paths. The 80.4% of trajectories originating from and passed over mainland China were defined as continental air (orange trajectories in Figure S1), the 12.4% from the South China Sea represented marine air (blue trajectories), and the remaining 7.2% was close to the coastline and denoted coastal air (brown trajectories). The latter two are collectively referred to as noncontinental air in this study.

### 2.2 Measurement Techniques

A PM_1_ cyclone was installed at the entry point of the particulate matter sampling duct to preclude the entry of particles exceeding an aerodynamic diameter of 1 μm. An online measurement system was implemented to quantitatively measure OA markers, including OHDCA. It was the first commercially available TAG developed by Aerodyne Research Inc. in conjunction with the University of California, Berkeley. Detailed descriptions of the instrument can be found elsewhere.^12,27^ Briefly, the samples were collected onto a collection and thermal desorption cell. After the derivatization of polar compounds by *N*-methyl-*N*-(trimethylsilyl)­trifluoroacetamide, the samples were transferred to a focusing trap to expel the nontarget compounds. Then, the remaining species were analyzed by gas chromatography coupled to time-of-flight mass spectrometry. We set a relatively long sampling duration (90 min) to ensure that the mass loading of many OA markers was high enough to be analyzed. Besides, the sample treatment, sample transfer, and other intermediate steps occupied an additional 30 min. The sample analysis was synchronized with the collection of the next sample. Therefore, a complete TAG cycle had a duration of 2 h, and the bihourly data represented the average over the 90 min sampling period every 2 h.

The OA markers were identified based on their retention times and ion fragments followed by peak fitting and integration and response-to-concentration conversion (calibration). A fixed amount of internal standard (IS) mixture was injected onto each sample and subjected to exactly the same treatment, transfer, and analysis procedures as ambient samples. The mixture was composed of dozens of deuterated or ^13^C-containing compounds in a wide range of volatility, chemically resembling but different from the target compounds.^12^ This was to track and correct for any changes in the instrument sensitivity and retention time drift. The response curves were made by analyzing authentic or surrogate standards at five different concentrations in the same manner as that for ambient samples. Table S1 lists the base ions, retention times, internal standards, coefficients of determination (*R* ^2^) for calibration curves, and average concentrations of the selected species. The quantitatively analyzed OHDCA species included malic acid, citramalic acid, 2-hydroxyglutaric acid, and tartaric acid.

High-resolution time-of-flight aerosol mass spectrometry (HR-ToF-AMS, hereinafter referred to as AMS) was utilized to measure the nonrefractory components in PM_1_, including total organic matter (PM_1_-OM), sulfate, nitrate, ammonium, and chloride, at a 5 min resolution. More details about the instrument and its applications were provided before.^21,28^ The number concentrations of particles in the size range of 10.9–514 nm were measured using a scanning mobility particle sizer (SMPS). The mixing ratios of O_3_, NO_2_, nitric oxide (NO), and carbon monoxide (CO) were continuously detected by the commercial instruments (Table S2). The average concentration was 40 ± 11 ppb (mean ± 95% confidence interval, same below) for O_3_, 0.7 ± 0.9 ppb for NO, 3.7 ± 4.7 ppb for NO_2_, and 225 ± 41 ppb for CO. Temperature (Temp) and relative humidity (RH) were monitored using a mini meteorological station, with the average values of 18.0 ± 3.7 °C and 58 ± 18%, respectively. The other meteorological data, such as wind direction, wind speed, and solar radiation, were obtained from the Hong Kong Observatory.

The aerosol liquid water content (LWC) was calculated using the thermodynamic model ISORROPIA-II, and the reverse mode was selected. The input data included sulfate, nitrate, ammonium, and chloride, measured by the AMS, temperature, and RH. The aerosol surface area (Sa) was estimated using the SMPS data with the assumption of spherical shape for all the particles and the consideration of hygroscopic growth as a function of RH.^29^

### 2.3 Random Forest Model Construction and Interpretation

Machine learning (ML) has the potential to reveal nonlinear relationships between variables. The random forest (RF) was used to identify the main factors influencing malic acid (a representative of OHDCA) concentrations. To enhance the robustness of the results, the observational data obtained in an earlier sampling campaign at a coastal background site (Hok Tsui) was combined with the TMS data. This combination was based on the fact that there were few anthropogenic emissions around both sites and that the two measurements were conducted using the same instruments by us. The Hok Tsui data was presented and discussed in a previous paper.^12^ Except for the Sa, the two sets of data were highly consistent in terms of analytical techniques and species detected. The SMPS used in the two field campaigns differed, leading to a discrepancy in the scanning range of the particle size distribution. Given the strong correlation between Sa and PM_1_ (Figure S2), we used the concentration of PM_1_ instead of Sa as an input of the RF model. Besides, the LWC data was incomplete in the Hok Tsui dataset, owing to a long period of AMS maintenance. With sulfate, nitrate in fine particulate matter (PM_2.5_), and other parameters as input, the RF was employed to simulate the missing LWC data at Hok Tsui. The predicted LWC agreed moderately with the calculated values, with an *R* ^2^ of 0.53 (Figure S3).

The variables were selected based on prior knowledge and the results of this study that imply relationships between malic acid and influencing factors (see Section 3.2). We selected the OA markers that might reflect potential precursors or formation pathways of malic acid, such as 2,3-dihydroxy-4-oxopentanoic acid (DHOPA) and 2-methylglyceric acid (2-MGA). Some factors indicating chemical processes were also adopted: LWC and sulfate for aqueous processes, O_*x*_ for photochemical processes, and PM_1_ (proxy for Sa) to reflect the role of particle surfaces. Additionally, the temperature was selected due to its influence on a wide range of chemical reactions. The physical implications of these variables are listed in Table S3. Next, we tested the model with other variables, such as solar radiation, wind direction, wind speed, gaseous pollutants (e.g., CO), and OA markers (e.g., levoglucosan, mannosan, palmitic acid, and fructose). However, the model performance did not improve, as indicated by the decrease in *R* ^2^ and relatively low importance for these variables (Figure S4). To test the impact of HT data on the results, ML was trained on the combined dataset and the TMS data only. We did not see significant differences in the ranking of the relative importance of the variables. However, due to significant underpredictions for the three highest concentrations and an overprediction for one low concentration, the model trained on TMS data alone showed a much worse performance in replicating the observed malic acid concentrations, with the *R* ^2^ of 0.32 compared to its counterpart’s *R* ^2^ of 0.79. Thus, we adopted the results of ML trained on the combined dataset.

A total of 394 sets of valid data were fed into the ML model (87 from TMS and 307 from Hok Tsui). The data were randomly divided into two subsets, and 80% of the data was used to train the model and the remaining 20% for validation. Hyperparameter tuning was performed using 5-fold cross-validation with a grid search approach. The key hyperparameters optimized included the number of trees (n_estimators), maximum tree depth (max_depth), and the minimum number of samples required to be at a leaf node (min_samples_leaf). The optimal hyperparameters were n_estimators = 600, max_depth = 30, and min_samples_leaf = 1. The model’s accuracy was confirmed using 10-fold cross-validation. As shown in Figure S3, the model performance is reasonably good, with the *R* ^2^, root-mean-square error (RMSE), and mean absolute error (MAE) of 0.79, 36.16, and 24.93, respectively. The Shapley additive explanation (SHAP) values were employed to quantify the contributions of independent variables to the variations of malic acid concentrations, which has been widely adopted in environmental research.^30–32^ The partial dependence plots (PDPs), offering an intuitive visualization of the factor effects with the other factors being held constant,^33^ were employed to visualize the relationships between the predicted malic acid concentrations and several pairs of two specific variables. The RF model was implemented in Python 3.9 using scikit-learn version 1.6.0 and shap v0.46.0 packages.

## 3 Results and Discussion

### 3.1 PM_1_-OM and OHDCA in Different Air Masses

Among all the PM_1_ components, the total organic matter ranked the first in concentrations at the TMS site (3.2 ± 0.3 μg/m^3^), which was even higher than the sum of sulfate (1.3 ± 0.1 μg/m^3^), ammonium (0.7 ± 0.1 μg/m^3^), nitrate (0.5 ± 0.1 μg/m^3^), and chloride (0.027 ± 0.004 μg/m^3^). The mass fraction of PM_1_-OM was up to 56.8%, significantly higher than the 43.8% at a regional background site and 47.1% at an urban site in HK.^21^ Although comparable to the 57.7% at a roadside site, large differences in organic compositions were to be expected as vehicle and cooking emissions were the dominant sources of PM_1_-OM at the roadside site.^28^ The average total concentration of all the detected OHDCA species was 185.1 ± 63.6 ng m^–3^ over the sampling period, with the median and maximum value of 91.0 and 1825 ng m^–3^, respectively. Among the detected OHDCA species, tartaric acid had the highest concentration (160.2 ± 53.4 ng m^–3^), followed by malic acid (18.6 ± 7.9 ng m^–3^), 2-hydroxyglutaric acid (3.7 ± 1.6 ng m^–3^), and citramalic acid (1.3 ± 0.6 ng m^–3^). Referring to a comprehensive global comparison of malic acid concentrations from a previous study,^22^ the malic acid concentration we observed was at the lower end of the reported range. The difference compared to previous measurements in HK was likely due to variations in meteorological conditions, such as lower temperatures and relative humidity than the 2020 HT measurements.^22^ The variations of the OHDCA species were highly consistent with each other with a *R* ^2^ of 0.900–0.996 (Figure S5). To facilitate discussion, we focus below on the total OHDCA or the most studied malic acid.

Figure 1 shows the time series of the selected OHDCA species and other selected parameters. It was found that the PM_1_-OM levels were the highest in the marine air (3.8 ± 1.3 μg m^–3^), followed by that in the most common continental air (3.3 ± 0.4 μg m^–3^) and coastal air (1.8 ± 0.4 μg m^–3^). Similarly, the concentrations of some SOA tracers, such as DHOPA, 2-MGA, 2-methyltetrols (2-MTs), and 3-hydroxy-4,4-dimethylglutaric acid (HDMGA), were also significantly increased in the marine air (Figure S6 and Table S4). With an average concentration of up to 554 ± 375 ng m^–3^, the marine air also witnessed the highest level of OHDCA for the entire sampling period at 4:00 am on November 7. This was in contrast to the prevailing notion that marine air is relatively clean. In fact, the marine air descended and passed over the urban areas of HK before arriving at the site (Figure S7). Sea-land breezes might also be involved in this process and brought secondary air pollutants formed in the offshore areas during the day to the site (Figure S8). These mesoscale circulations were indirectly proved by the elevated levels of many primary (e.g., CO, NO_*x*_, levoglucosan, and mannosan) and secondary air pollutants (e.g., O_3_, nitrate, and sulfate) in the case with the highest OHDCA. Thus, the emissions in the urban areas might have replenished the precursors of SOA including OHDCA (see Section 3.3). Besides, the wind speed was the lowest in the marine air case (0.4 ± 0.2 m s^–1^), which tended to facilitate air pollutant accumulation. The highest temperature (22.9 ± 1.6 °C) could also contribute to biogenic emissions and subsequent formation of SOA, including 2-MGA, 2-MTs, and HDMGA, which might be further enhanced by the highest LWC. Our recent studies indeed suggested that the high levels of OHDCA observed at Hok Tsui were formed through aqueous photooxidation processes.^12^ ^,^ ^22^

**Figure 1:**
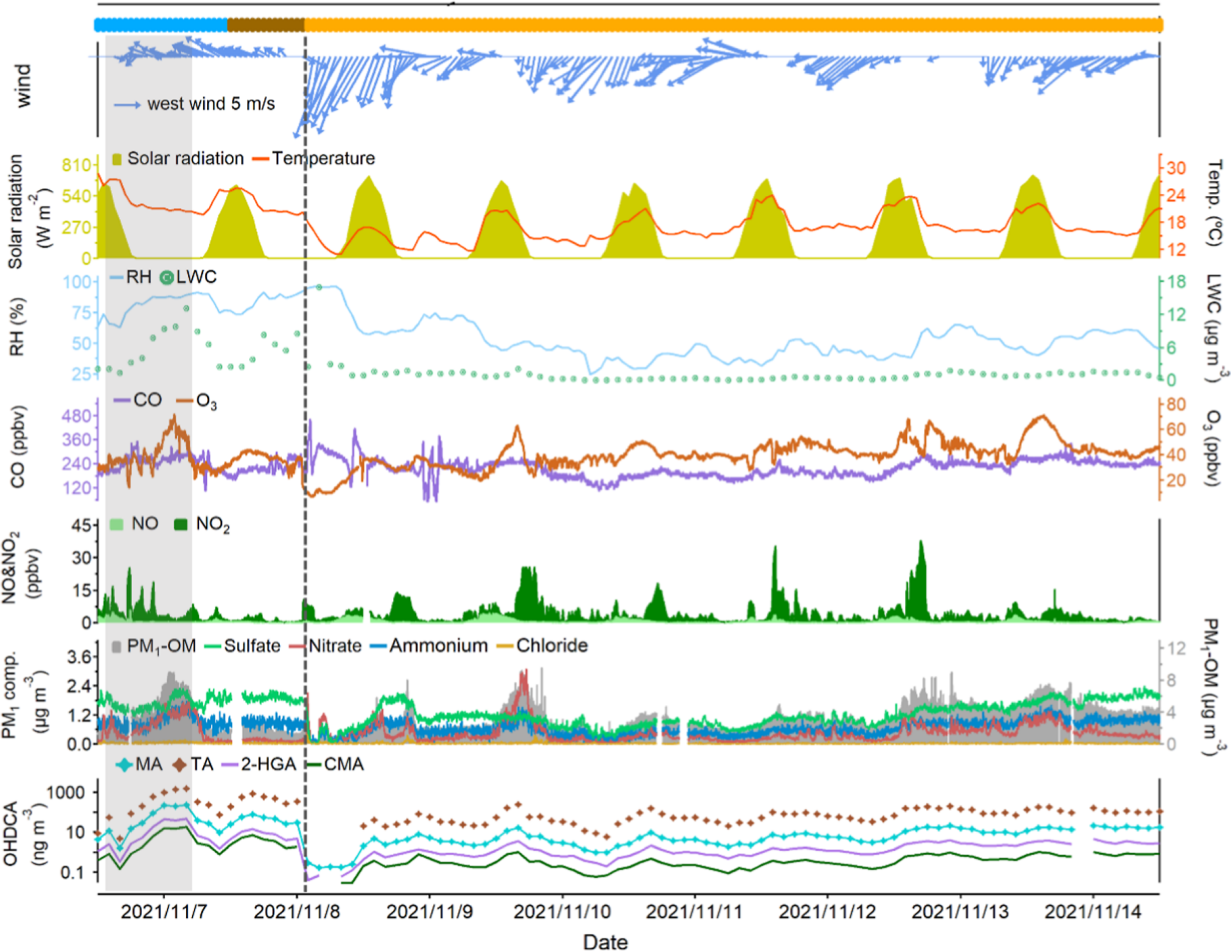
Time series of OHDCA species, nonrefractory PM_1_ components, trace gases, and meteorological parameters. Blue, brown, and orange bars at the top represent the marine, coastal, and continental air, respectively. The case with the highest levels of OHDCA is shaded, and the vertical dashed line indicates the arrival of the cold front. Missing data is due to instrument maintenance.

The arrival of a cold front in the early hours of November 8 brought the continental air from the mainland to HK, resulting in a significant decline in temperature, RH, and LWC lasting until the end of the sampling. Although the high levels of primary air pollutants (e.g., NO_*x*_) implied sufficient anthropogenic precursors for SOA formation, the concentrations of many SOA tracers including the anthropogenic DHOPA were notably reduced, indicating that SOA formation was suppressed by the unfavorable meteorological conditions. The concentration of total OHDCA was also the lowest in continental air (89.5 ± 14.1 ng m^–3^). The coastal air, as a transition between the marine air and continental air, featured the highest RH and strongest ultraviolet. In contrast to the high concentrations of sulfate and ammonium, PM_1_-OM exhibited the lowest levels in the coastal air (1.8 ± 0.4 ng m^–3^). Despite this, the concentration of OHDCA (535.4 ± 189.0 ng m^–3^) was comparable to that in the marine air. Likewise, aqueous processes might also play an important role in the formation of OHDCA in the coastal air, as implied by the high levels of LWC and sulfate (Table S4).

### 3.2 Potential Factors Influencing OHDCA Formation

To preliminarily identify the factors that influence the formation of OHDCA, we performed correlation analysis between malic acid and various OA tracers and several factors regulating chemical reactions. The data of marine and coastal samples that showed similar correlations with the influencing factors were combined. Figure 2 shows the correlations excluding three samples with exceptionally high malic acid concentrations observed in the marine air case. As discussed above, these elevated concentrations might result from the combination of aqueous photochemical processes and mesoscale circulation, which is further confirmed in Section 3.3. The correlations with the inclusion of the three samples are shown in Figure S9. Moderate to good correlations were identified between malic acid and several SOA tracers, including DHOPA, 2-MGA, HDMGA, and pinic acid (PA). DHOPA is generally formed from aromatics, and based on such correlation, a previous study indicated that aromatics were the potential precursors of OHDCA.^22^ PA and HDMGA are the earlier and later generation oxidation products of monoterpenes.^35–37^ Both 2-MGA and 2-MTs are isoprene SOA tracers, formed under high-NO_*x*_ and low-NO_*x*_ conditions, respectively.^38–40^ The correlations with PA, HDMGA, and 2-MGA suggested that the oxidation of monoterpenes and isoprene might also have contributed to the formation of malic acid. It was also likely that OHDCA had similar formation pathways to those of these SOA tracers.

**Figure 2:**
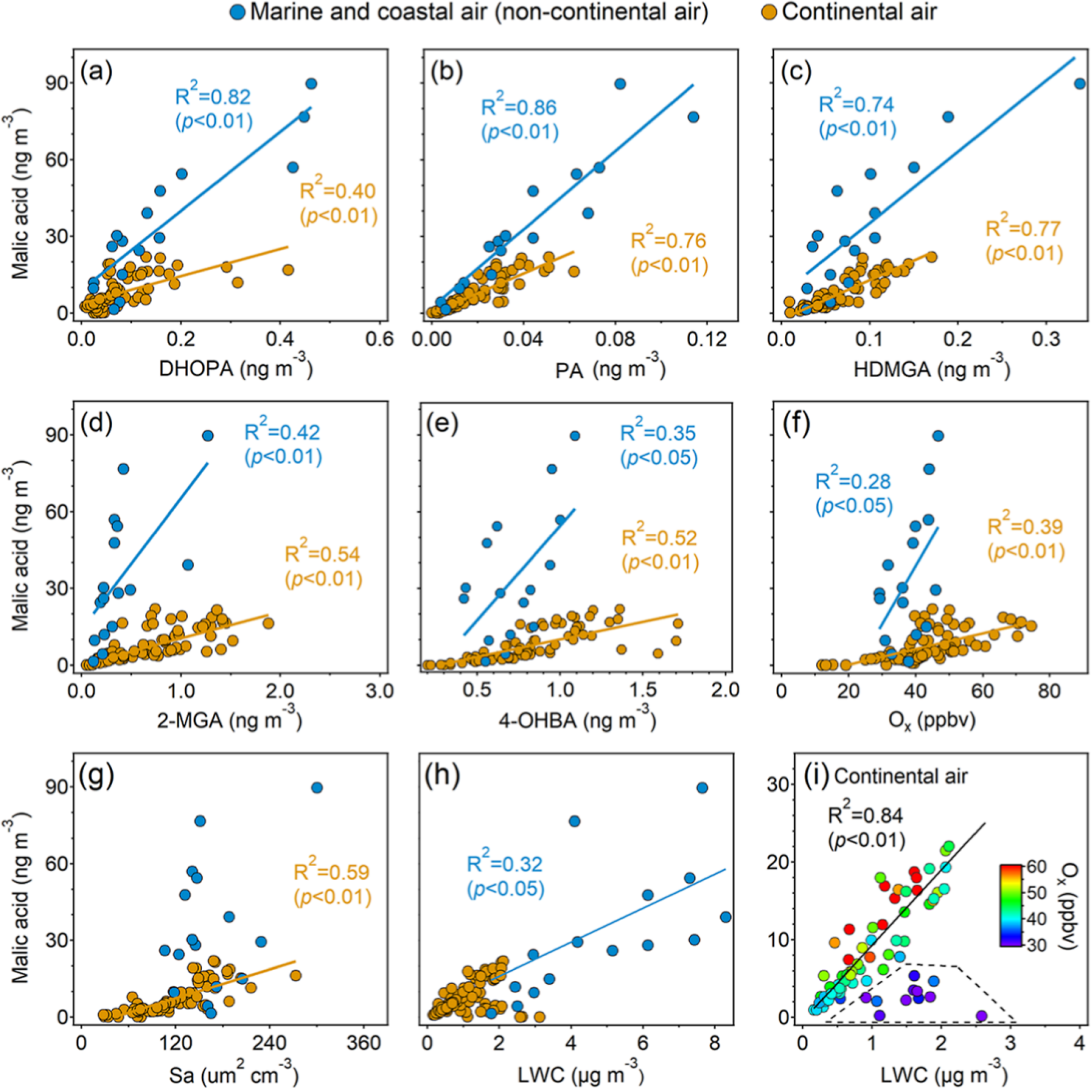
Correlations of malic acid with OA tracers and factors regulating chemical reactions excluding three exceptionally high values of malic acid: DHOPA (a), PA (b), HDMGA (c), 2-MGA (d), 4-OHBA (e), O_*x*_ (f), Sa (g), and LWC (h). Correlation with LWC in the continental air color-coded by O_*x*_ is shown in (i).

However, malic acid showed no relationship with 2-MTs, which might be due to the different formation pathways. In line with several recent observations in HK,^12,34,41^ the 2-MTs exhibited higher concentrations at night, in contrast to the photochemical patterns of malic acid and 2-MGA. The reasons for this unexpected diurnal pattern remain unclear. An exception occurred in the case of marine air, where synchronized variations of malic acid and 2-MTs were observed from the early afternoon to midnight. It is also worth noting that malic acid correlated moderately with 4-OHBA, a tracer of biomass burning (BB) and BB-derived SOA. However, there was no correlation between malic acid and levoglucosan or mannosan. Previous studies^42,43^ have shown that 4-OHBA and vanillic acid are the products of lignin combustion, while levoglucosan and mannosan originate from the burning of cellulose and hemicellulose. Although plastic incineration also releases 4-OHBA,^44^ other coemitted tracers were not detected and there existed a strong correlation between 4-OHBA and vanillic acid in the continental air. Hence, the moderate correlation of malic acid with 4-OHBA implied the contributions of some types of BB, which remain to be clarified.

Moreover, malic acid also showed close relationships with O_*x*_, Sa, and LWC. Correlations with O_*x*_ and LWC (or RH) were regarded as a sign of aqueous photochemical formation of the studied species.^21,45,46^ The correlation between malic acid and LWC in continental air was undermined by some samples with low levels of O_*x*_. Excluding these samples would substantially increase *R* ^2^ from 0.30 to 0.83. This could be explained by the possibility that the aqueous formation of malic acid requires certain levels of oxidants, which is supported by the synergistic effect of O_*x*_ and LWC in malic acid formation, as discussed in Section 3.3. The aerosol surface provides a unique environment for chemical reactions.^47^ The correlation of malic acid with Sa indicated that the aerosol surface offered favorable conditions for malic acid formation, such as surface adsorption of precursors and the provision of water and oxidants. It is worth noting that the three samples with malic acid concentrations higher than 200 ng m^–3^ also witnessed proportionally high levels of O_*x*_, Sa, and LWC. Therefore, these factors were likely to enhance malic acid formation, even for the three nighttime samples. Further, the slopes of the regression lines for the noncontinental air were consistently higher than those for the continental air. We suspect that there were other reasons besides the aforementioned factors that made malic acid formation more efficient in the marine and coastal air.

### 3.3 Relative Importance and Combined Effects of Influencing Factors

To understand in a more holistic way the relative importance of the factors influencing malic acid concentrations, we employed the RF algorithm and calculated the SHAP values of the factors (Figure 3). Based on the mean absolute SHAP values at TMS (Figure 3a), it appears that DHOPA ranked first in affecting malic acid concentrations due to shared precursors (aromatics) or similar formation pathways with malic acid. Likewise, the relative importance of PA and HDMGA (monoterpenes SOA tracers) was also high. The lower but still positive SHAP values of 2-MGA implied a weak connection with isoprene oxidation under high-NO_*x*_ environments. Moreover, the LWC and sulfate ranked second and fourth in the absolute SHAP values, respectively, reiterating the important role of aqueous processes in malic acid formation. Although less significant, the positive effects of photooxidation, particle surface area, and temperature on malic acid concentrations also existed, as suggested by the SHAP values of O_*x*_, PM_1_, and temperature (Figure 3b).

**Figure 3:**
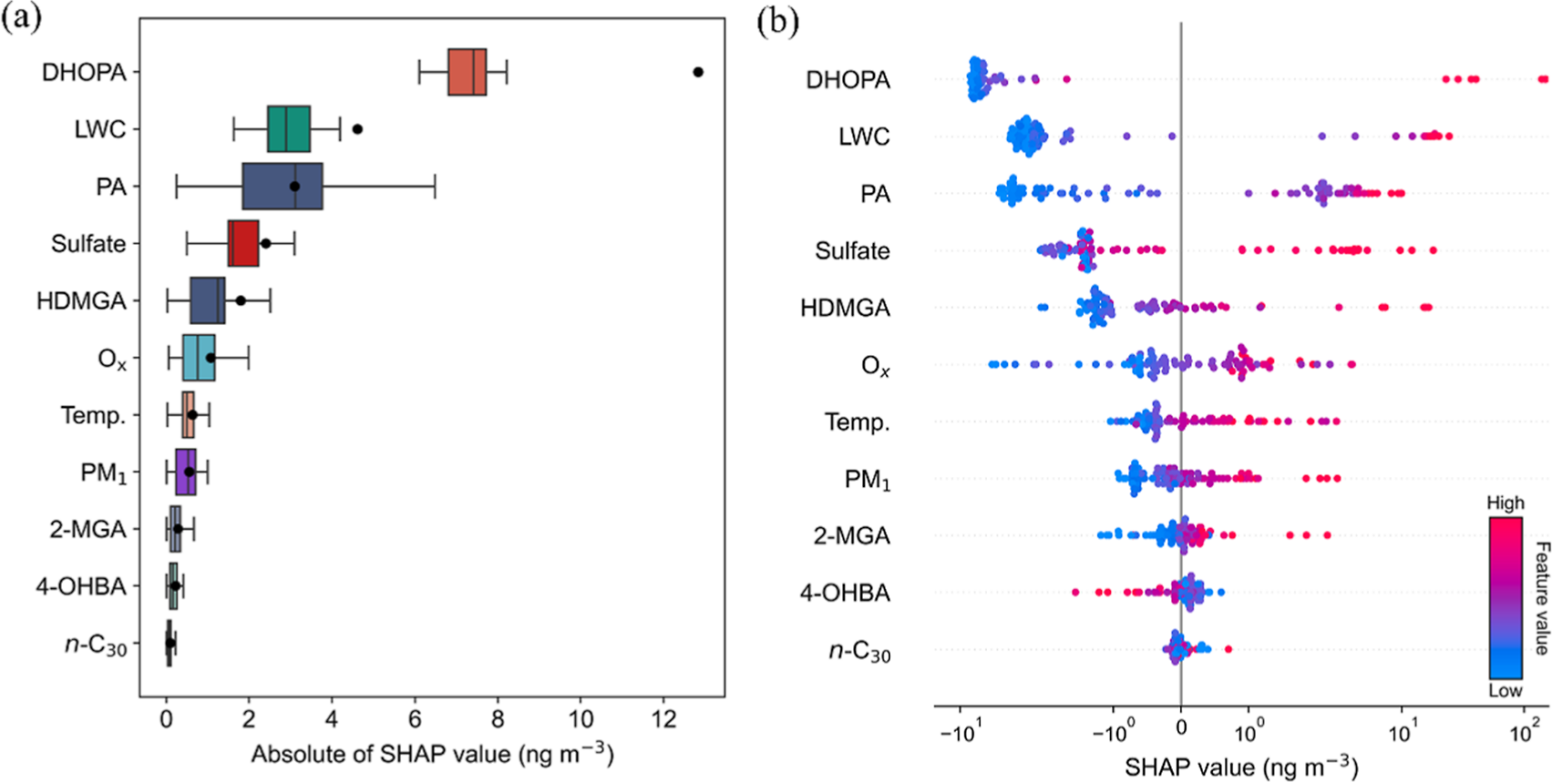
Factors influencing malic acid concentrations at TMS ranked by mean absolute SHAP values (a) and summary plot of SHAP values of each factor color-coded by feature values (b).

Next, PDPs were employed to show the combined effects of two of the factors on malic acid concentrations, while the other factors were fixed (Figure 4). Using the pair of LWC and O_*x*_ for an example, the malic acid concentration increased with higher levels of LWC and O_*x*_, and the increase was particularly pronounced when the LWC and O_*x*_ rose simultaneously. This provides strong evidence for aqueous photochemical formation of malic acid, a process that was proposed but not fully substantiated previously.^22^ Besides, a similar synergy was also identified between LWC and temperature, as well as between O_*x*_ and temperature. Sulfate, another indicator of aqueous processes, also increased malic acid concentrations alongside the levels of O_*x*_, temperature, and PM_1_, with the effect being particularly pronounced at higher sulfate levels. The inconsistent effects of LWC and sulfate at their low levels might be due to their different thresholds for indicating aqueous processes and/or stimulating malic acid formation. Particle surface area (represented by PM_1_) also showed some synergies with the other factors, which however were weaker, as indicated by the contours being more parallel to the PM_1_ axis (Figure 4). Moreover, the responses of the malic acid concentration to the combination of the above factors and precursor proxies were also examined, as shown in Figure S10. The synergy was also discernible, except that the effect of DHOPA was much less dependent upon the factors including LWC, O_*x*_, PM_1_, and temperature with unknown reasons. Despite this, there was still an enhancement of malic acid concentration with an increase in LWC or temperature in the high-DHOPA zone, corresponding to the marine air case with the highest levels of malic acid for the entire sampling period.

**Figure 4:**
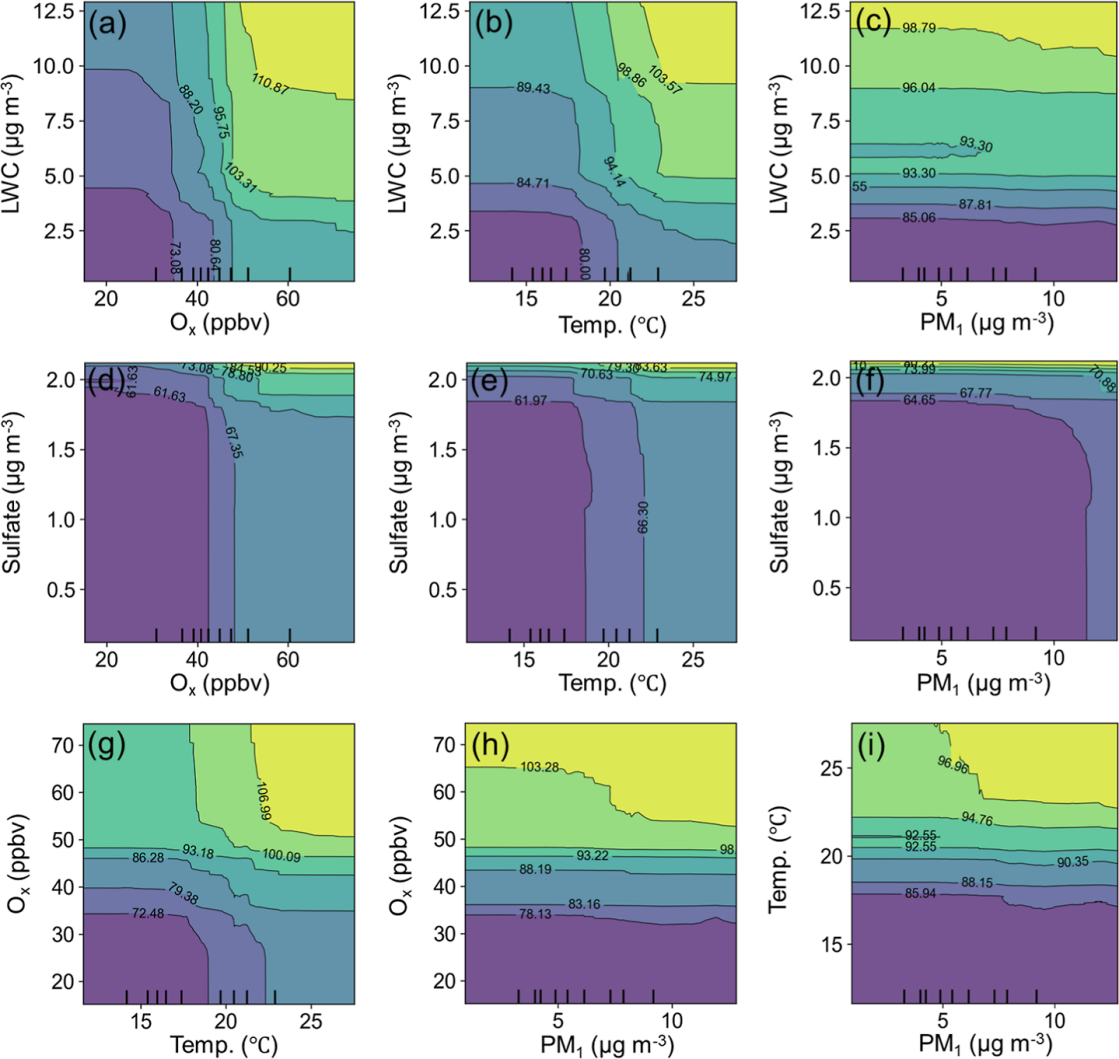
Synergistic effects of a pair of two factors on malic acid concentrations: LWC–O_*x*_ (a); LWC–temperature (b); LWC–PM_1_ (c); sulfate–O_*x*_ (d); sulfate–temperature (e); sulfate–PM_1_ (f); O_*x*_–temperature (g); O_*x*_–PM_1_ (h); temperature–PM_1_ (i).

Given the notable difference in malic acid concentrations between the continental and noncontinental air, we further compared the relative importance of the influencing factors between the two types of air masses by calculating the differences in SHAP values. Figure 5a shows the stacked plot of the differences. It was found that DHOPA contributed the most (55.6%, 31.0 ng m^–3^) to the increase in malic acid concentrations in the noncontinental air, followed by LWC (24.1%, 13.4 ng m^–3^), PA (7.2%, 4.0 ng m^–3^), HDMGA (6.6%, 3.7 ng m^–3^), and sulfate (4.0%, 2.3 ng m^–3^). Therefore, it was likely that aqueous-phase oxidation of aromatics and monoterpenes, and/or the similar formation pathways of their resulting SOA, caused the increase in malic acid concentrations in the noncontinental air. While the effects of the other factors are understandable, the significant contribution of aromatics, if presented as precursors, was surprising. As discussed above, the noncontinental air masses with high levels of malic acid might have carried aged land-based air pollutants from offshore areas and passed through urban areas before arriving at the site. Namely, the critical role of aromatics could be explained by urban emissions and aging under mesoscale circulations. However, this did not necessarily mean that continental air lacked aromatic compounds. Instead, it was likely the unfavorable meteorological conditions (e.g., low relative humidity) that inhibited the oxidation efficiency of aromatics. Conversely, the SHAP value of O_*x*_ was higher by 0.8 ng m^–3^ in the continental air, indicating the more important role of photochemistry in malic acid formation in air from the inland.

**Figure 5:**
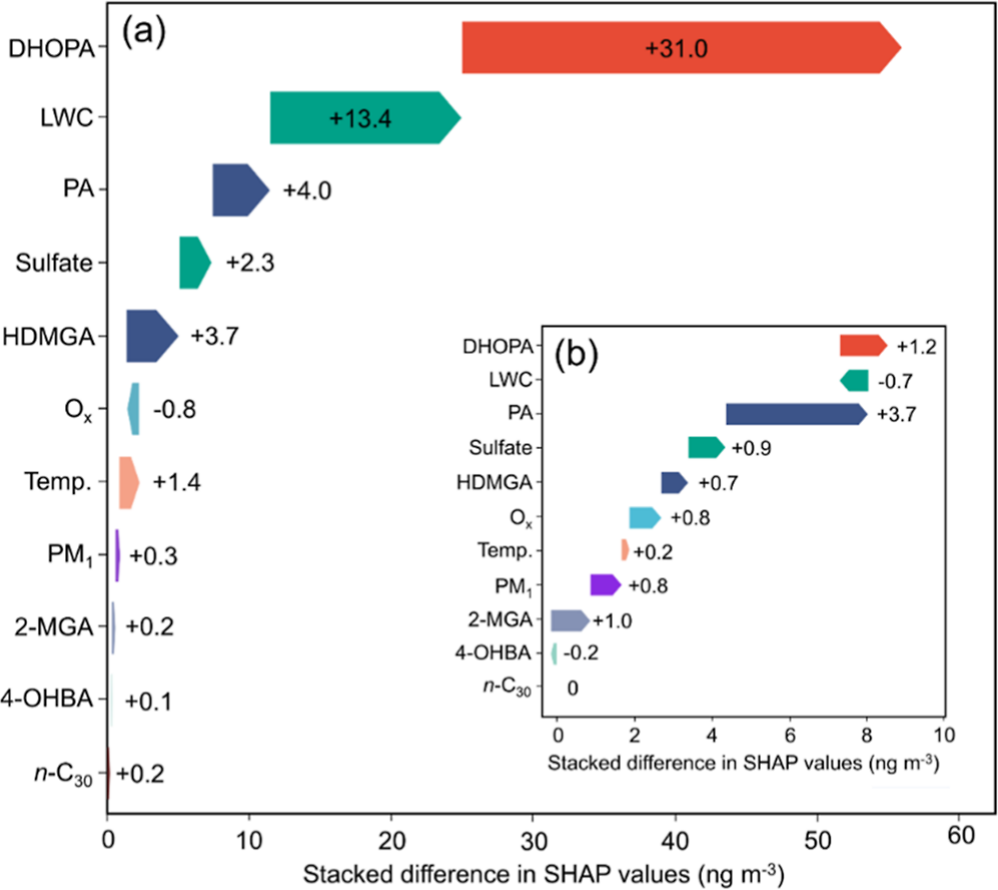
Differences in SHAP values of individual factors between the continental and noncontinental air (a), with the inset showing the differences between samples with malic acid concentrations above and below the 5th percentile in continental air (b).

We also examined the main factors leading to variations in malic acid concentrations in continental air by calculating the changes in SHAP values with those for malic acid concentrations below the fifth percentile as the reference. As shown in Figure 5b, the high values of continental malic acid were mainly associated with PA, DHOPA, and 2-MGA, demonstrating the mixed contributions of multiple precursors including monoterpenes, aromatics, and isoprene and/or similar formation pathways of malic acid with these SOA tracers. As isoprene SOA tracers, the ratio of 2-MGA to 2-MTs in the continental air was 11.2 times higher than in noncontinental air, which might explain the different effects of 2-MGA (a proxy for isoprene oxidation in NO_*x*_-rich environments) on malic acid concentrations in the two types of air masses. Additionally, the particle surface area (represented by PM_1_) and O_*x*_ also elevated malic acid concentrations. In contrast to the positive difference for sulfate, the difference in the SHAP value for LWC between samples above and below the fifth percentile was negative. Again, this could be due to the inconsistent thresholds of sulfate and LWC in indicating aqueous processes. Given that sulfate in the continental air had much lower concentrations and could be formed through nonaqueous pathways, aqueous formation seemed not to be a primary factor leading to the increase in malic acid concentrations in the continental air.

### 3.4 Implications for Particle Growth

Particle growth significantly affects the climate effects of atmospheric particulate matter.^48,49^ Here, we focus on a special event, where the particles started to grow at ∼14:00, which happened to be the marine air case with the highest concentrations of the OHDCA concentrations. As illustrated in Figure 6, the geometric mean diameter (GMD) of particles increased from ∼50 nm at 16:00 to ∼110 nm (CCN-size scale) at 00:50 on the following day, corresponding to a growth rate of 6.5 nm/h. Consistently, the concentrations of total organics, nitrate, and ammonium in PM_1_ also increased significantly. In this period, GMD showed excellent correlations with PM_1_-OM and nitrate and moderate correlations with the other PM_1_ components (Figure S11). Further, it correlated fairly well (*R* ^2^ = 0.88) with the CO_2_^+^ ion (*m*/*z* 44) measured by the AMS, an indicator of SOA.^50^ This event also featured a striking rise in the level of OHDCA and the highest levels of OHDCA concentrations throughout the sampling period. Moreover, a close relationship between the GMD and malic acid with an *R* ^2^ of 0.93 was identified. There also existed similar correlations between the GMD and some other SOA tracers, higher than the correlations with primary OA tracers (Figure S12). Therefore, this relatively rare particle growth might have been partially enhanced by particulate organic matter including OHDCA-like SOA. It is noteworthy that the temperature decreased from the afternoon to midnight, which was conducive to gas-to-particle partitioning of semivolatile species, such as nitrate. However, the volatilities of OHDCA species were much lower, so their concentrations in PM_1_ were less dependent upon temperature. The increase in the concentrations of OHDCA was likely a result of chemical formation and/or transport, which potentially contributed to the particle growth.

**Figure 6:**
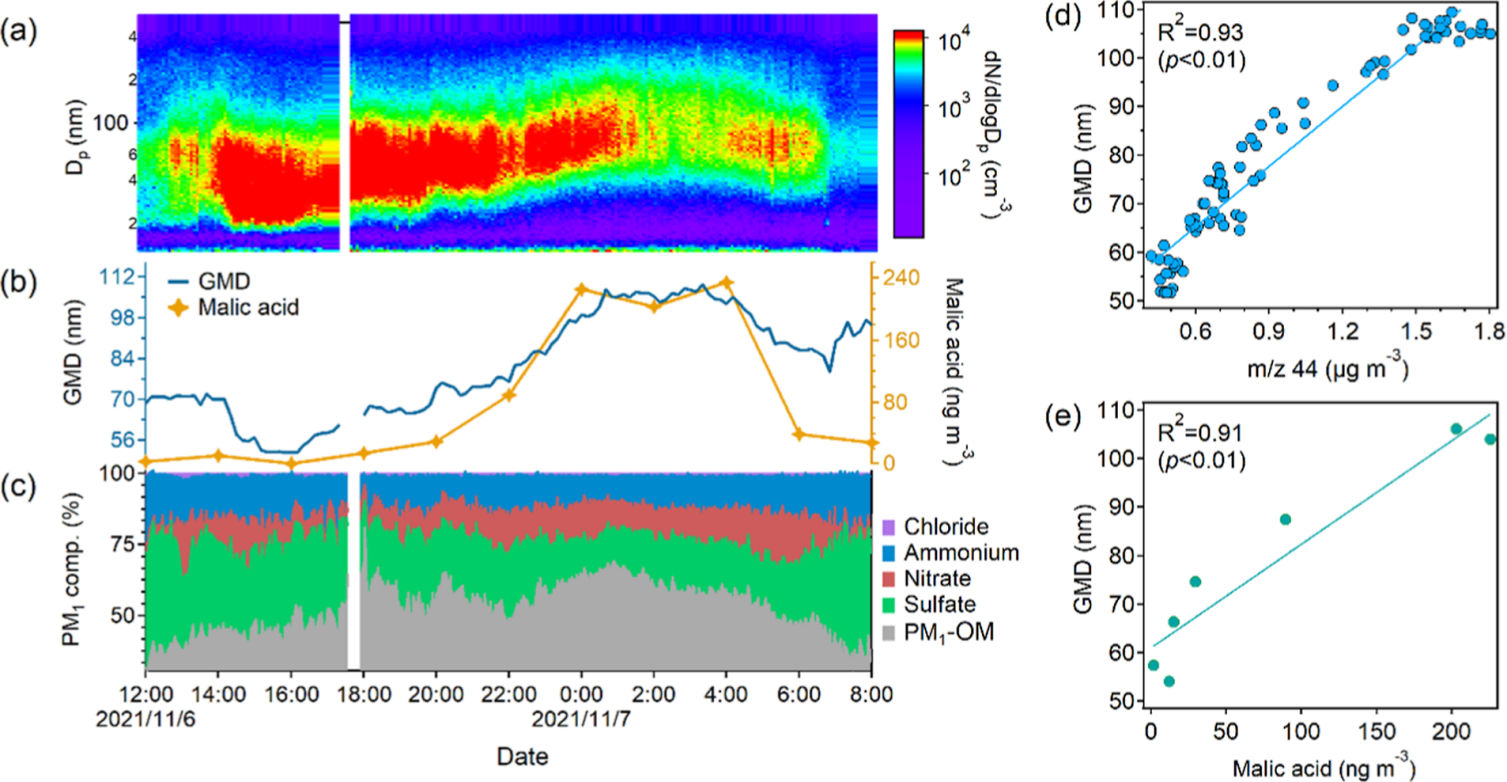
A particle growth event on November 6–7: evolutions of particle number distribution across size bins (a); GMD and malic acid (b); and fractions of PM_1_ components (c); and correlations of GMD with *m*/*z* 44 (d); and malic acid (e).

## 4 Conclusions

As a typical OA component, OHDCA has been detected at high concentrations in many regions around the world, including South China. The high proportions of hydrophilic groups make it a potential climate mediator. Studies identified various precursors and chemical processes that account for the formation of OHDCA. However, there has been a deficiency in a quantitative understanding of the relative importance and synergistic effects of these factors. In this study, we measured several OHDCA species at a higher-than-usual temporal resolution in an area with compounded air pollution sources. With the aid of ML, this study revealed the dominant contributions to malic acid (a representative of OHDCA) of aromatics and monoterpenes and/or similar formation pathways with their resulting SOA in the noncontinental air, which was intertwined with aqueous processes. These factors might also explain the highest concentrations of the OHDCA for the entire sampling period observed in the marine air. The leading role of aromatics, if presented as precursors, in the noncontinental air was counterintuitive, yet it seemed to be explained by mesoscale circulations. In addition to these factors (excluding LWC), isoprene, particle surface area, and O_*x*_ also contributed to the elevated levels of malic acid in the continental air. Moreover, ML also confirmed the synergistic effects of LWC (or sulfate), O_*x*_, and temperature on malic acid concentrations, indicating aqueous photochemical formation processes. Our results also shed light on the climate effects of the OHDCA, which likely facilitated the growth of atmospheric particles on the CCN-size scale. Despite the above, limitations also exist, mainly in the form of model uncertainties caused by the limited number of data points and variables. Future research should aim to improve the generalizability of ML results by utilizing more comprehensive data.

## Supplementary Material



## Data Availability

All the raw data are available upon request from the corresponding author Dr. Xiaopu Lyu.

## Notes

Site location; Sa-PM_1_ correlations; model performance evaluation; SHAP values for different combinations of variables; correlations between OHDCA species; time series of selected OA markers; backward trajectories in a special case; wind and temperature evolutions in a special case; correlations of malic acid with influencing factors; synergistic effects on malic acid concentrations; correlations of GMD with PM_1_ components; correlations of GMD with OA markers; identification and quantification metrics for OA markers; real-time measurements of trace gases; ML input variables; and comparisons between air flows (PDF)
