# Supplementary material for: Hydroxyl Dicarboxylic Acids at a Mountainous Site in Hong Kong: Formation Mechanisms and Implications for Particle Growth
Source: ACS Environ Au. 2025 Mar 14;5(3):277–86. doi: 10.1021/acsenvironau.4c00119 (PMC12100550; doi:10.1021/acsenvironau.4c00119)
Supplement: Supplementary file 1 [file vg4c00119_si_001.pdf]

Supporting Information of the manuscript

Hydroxyl dicarboxylic acids at a mountainous site in Hong Kong: formation mechanisms and implications for particle growth

Hongyong Li[1,2], Xiaopu Lyu[2*], Likun Xue[1*], Yunxi Huo[3], Tianshu Chen[3], Dawen Yao[4], Haoxian Lu[5], Beining Zhou[3], Hai Guo[3*]

[1] Environment Research Institute, Shandong University, Qingdao, China

[2] Department of Geography, Faculty of Social Sciences, Hong Kong Baptist University, Hong Kong

[3] Department of Civil and Environmental Engineering, The Hong Kong Polytechnic University, Hong Kong

[4]School of Intelligent Systems Engineering, Sun Yat-Sen University, Shenzhen, Guangdong, China

[5]Southern Marine Science and Engineering Guangdong Laboratory (Zhuhai), Guangdong, China

* Correspondence to Xiaopu Lyu (xiaopu_lyu@hkbu.edu.hk); Likun Xue (xuelikun@sdu.edu.cn); Hai Guo (hai.guo@polyu.edu.hk)

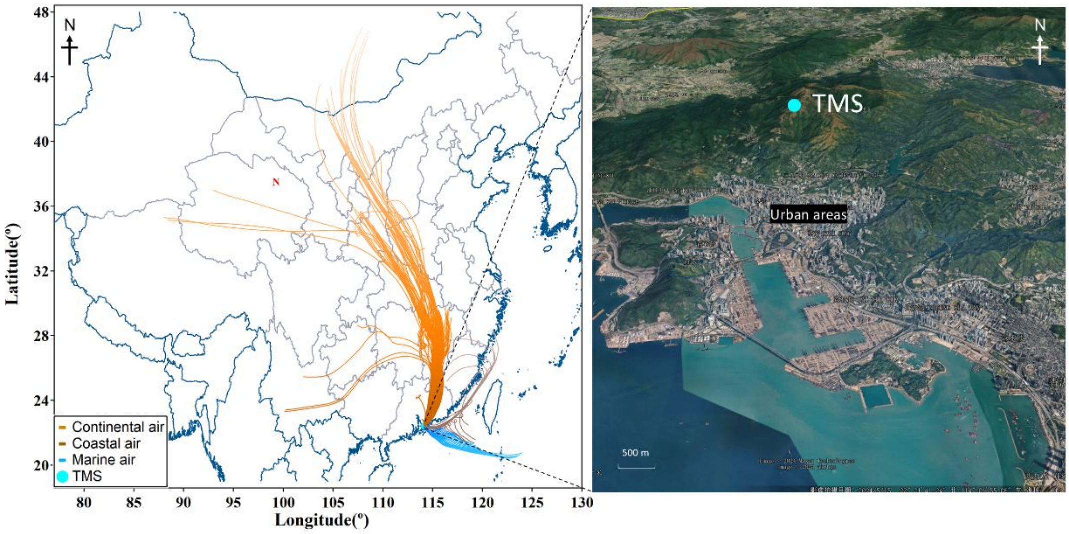

Figure S1. Location of the sampling site with 48-h backward trajectories of three types of air masses (left) and the satellite image of the study area (right).

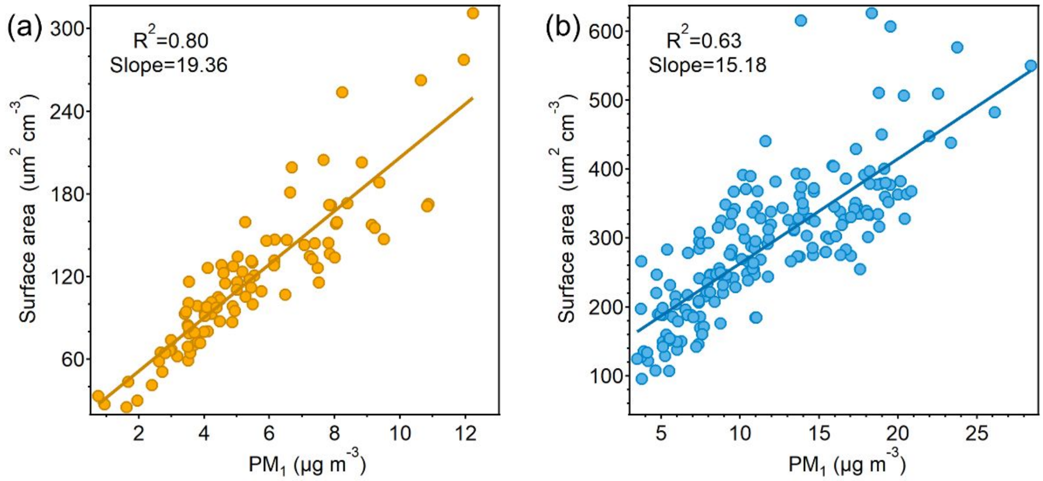

Figure S2. Correlation of particle surface area with PM_1 at TMS (a) and Hok Tsui (b).

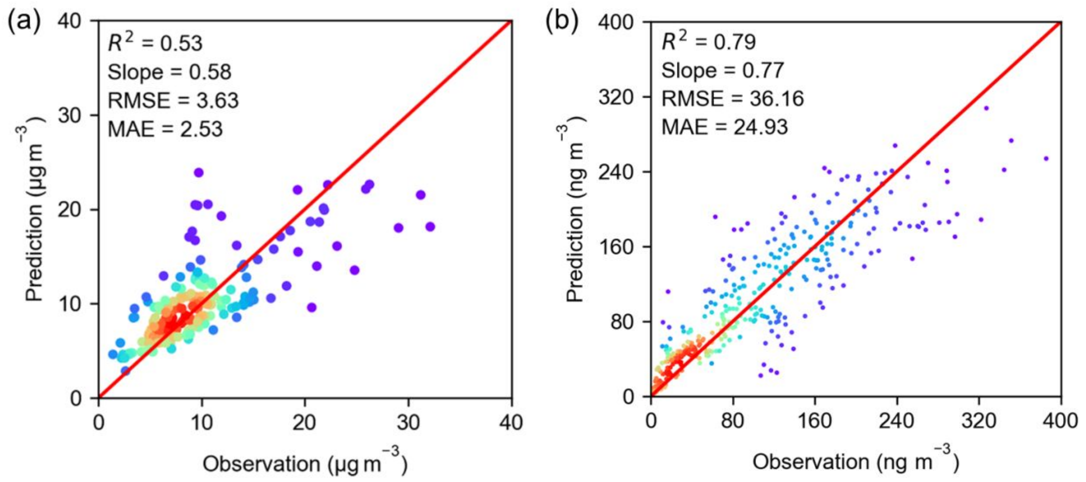

Figure S3. Performance of the RF model in predicting LWC at Hok Tsui (a) and malic acid at TMS and Hok Tsui (b). The red line indicates the 1:1 line, and the data points are color-coded by data frequency.

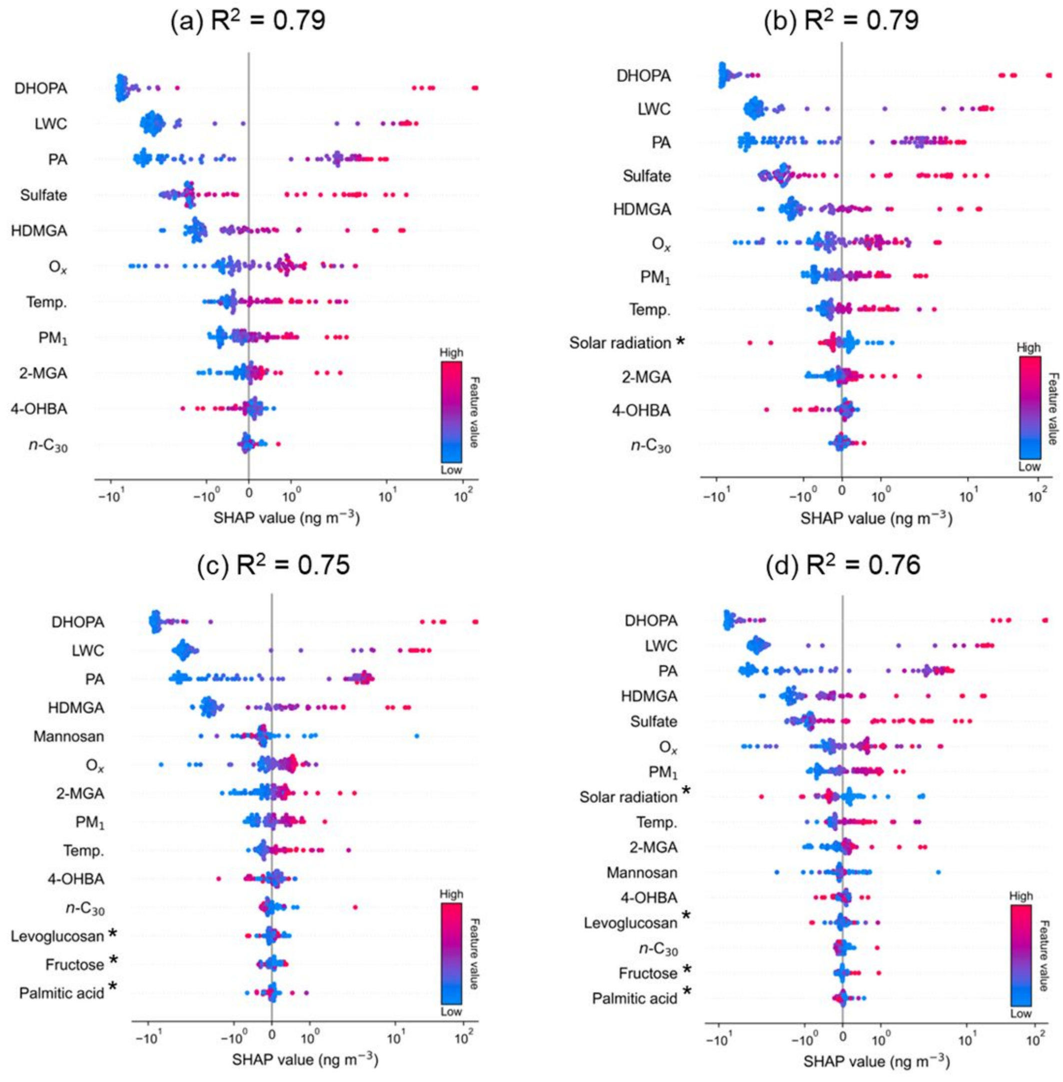

Figure S4. Summary plot of SHAP values color-coded by feature values for different combinations of variables. (a) represents the solution we adopted, and (b)-(d) show the results of adding different variables. R^2 indicates the level of agreement between the predicted and observed malic acid concentrations.

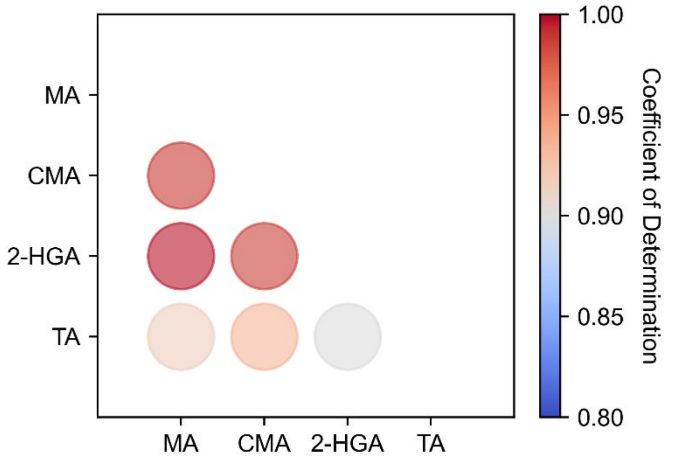

Figure S5. Coefficient of determination (R^2) for the correlations between OHDCA species.

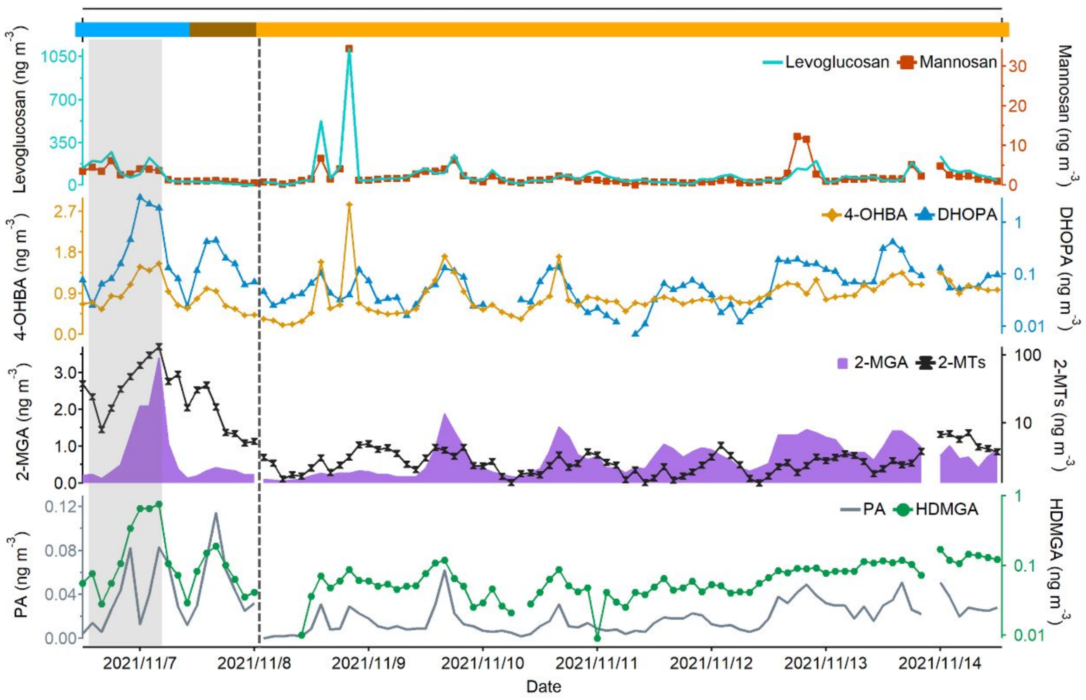

Figure S6. Same as Figure 1, but for some other selected OA markers.

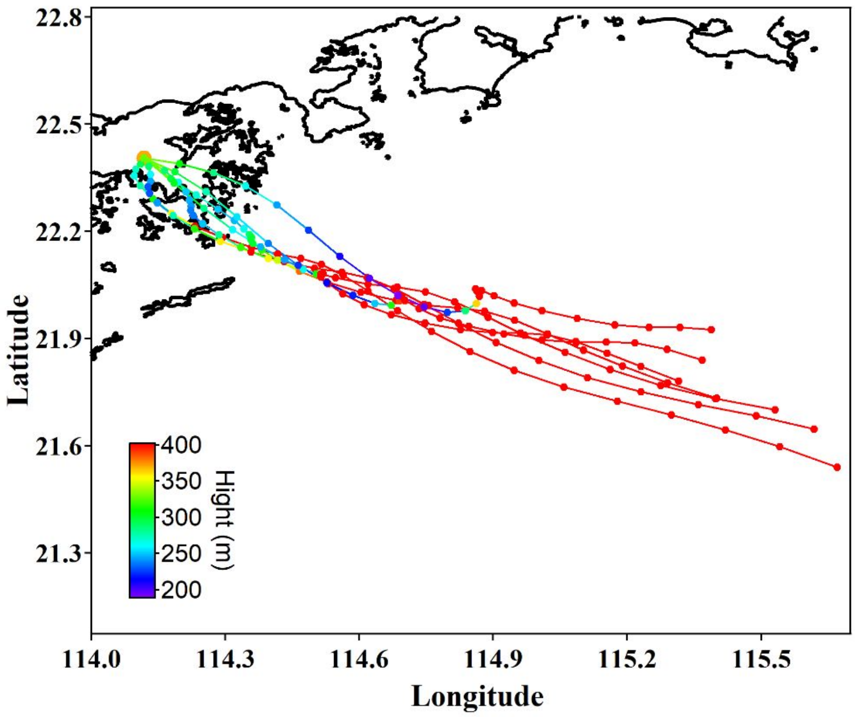

Figure S7. 24-hr backward trajectories arriving at TMS from 16:00 on November 6 to 04:00 on November 7 color-coded by altitude.

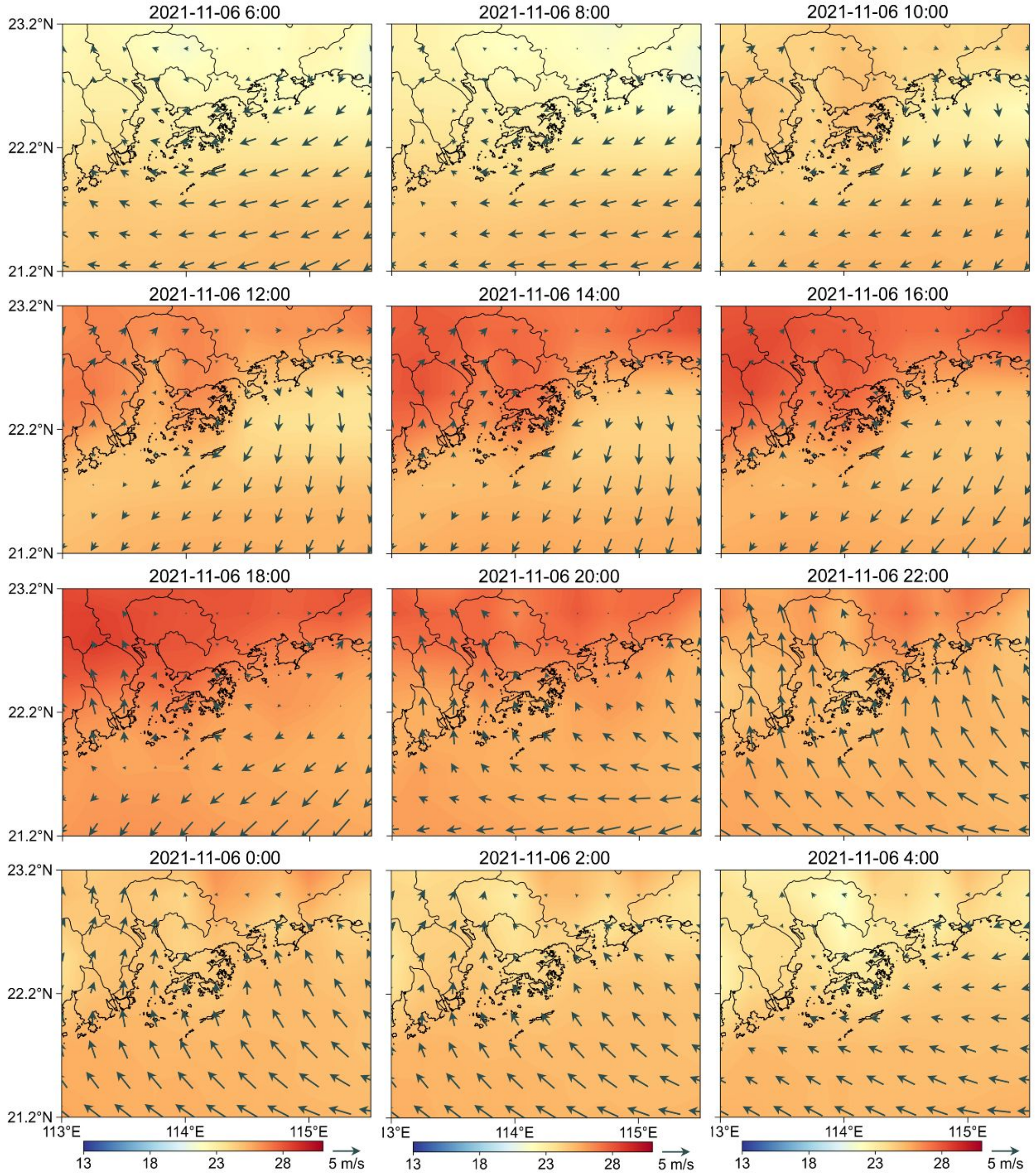

Figure S8. Evolution of temperature (color) and wind fields (arrows) over South China from 6:00 on November 6 to 4:00 on November 7.

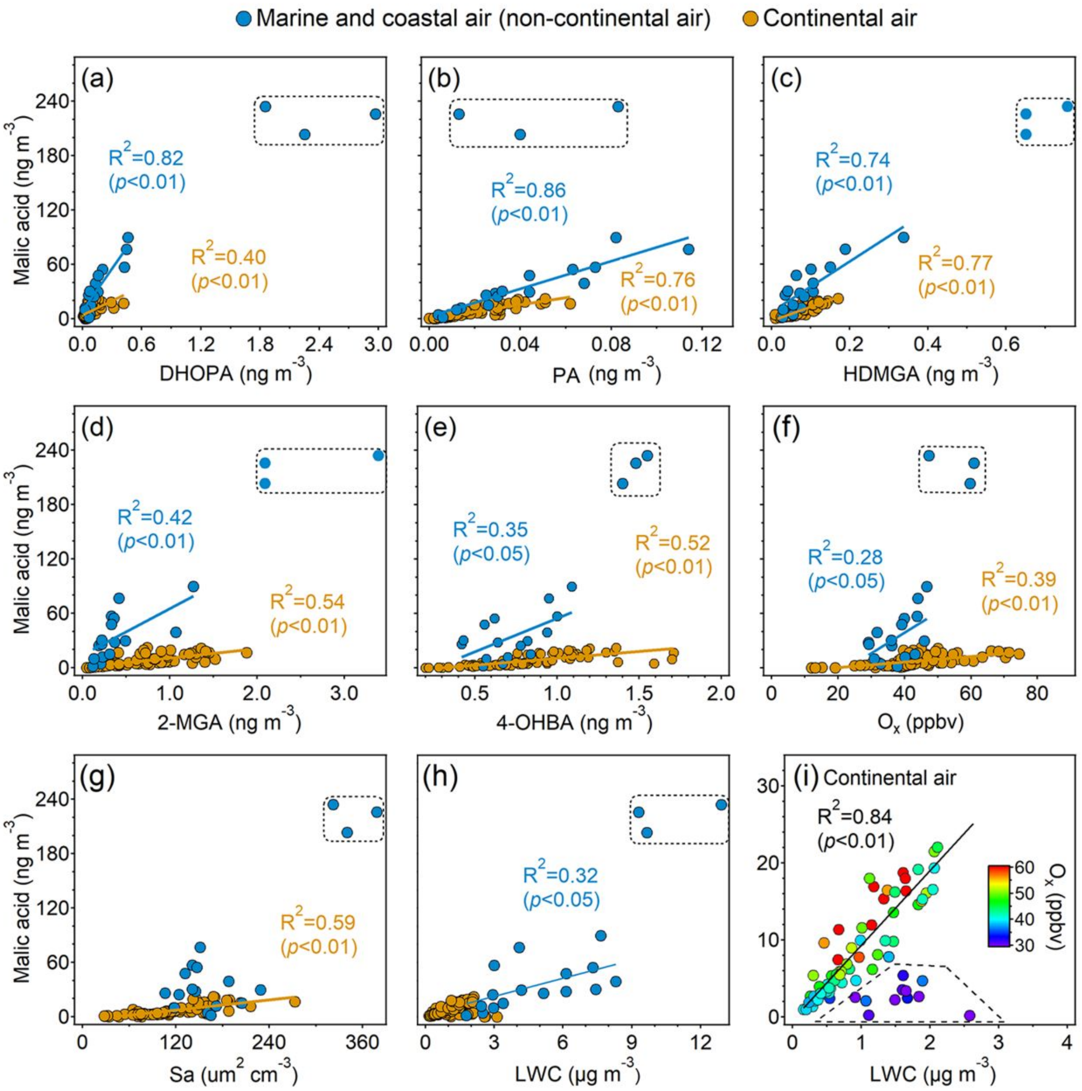

Figure S9. Same as Figure 2, but including the three samples with exceptionally high malic acid concentrations (inside the dashed box).

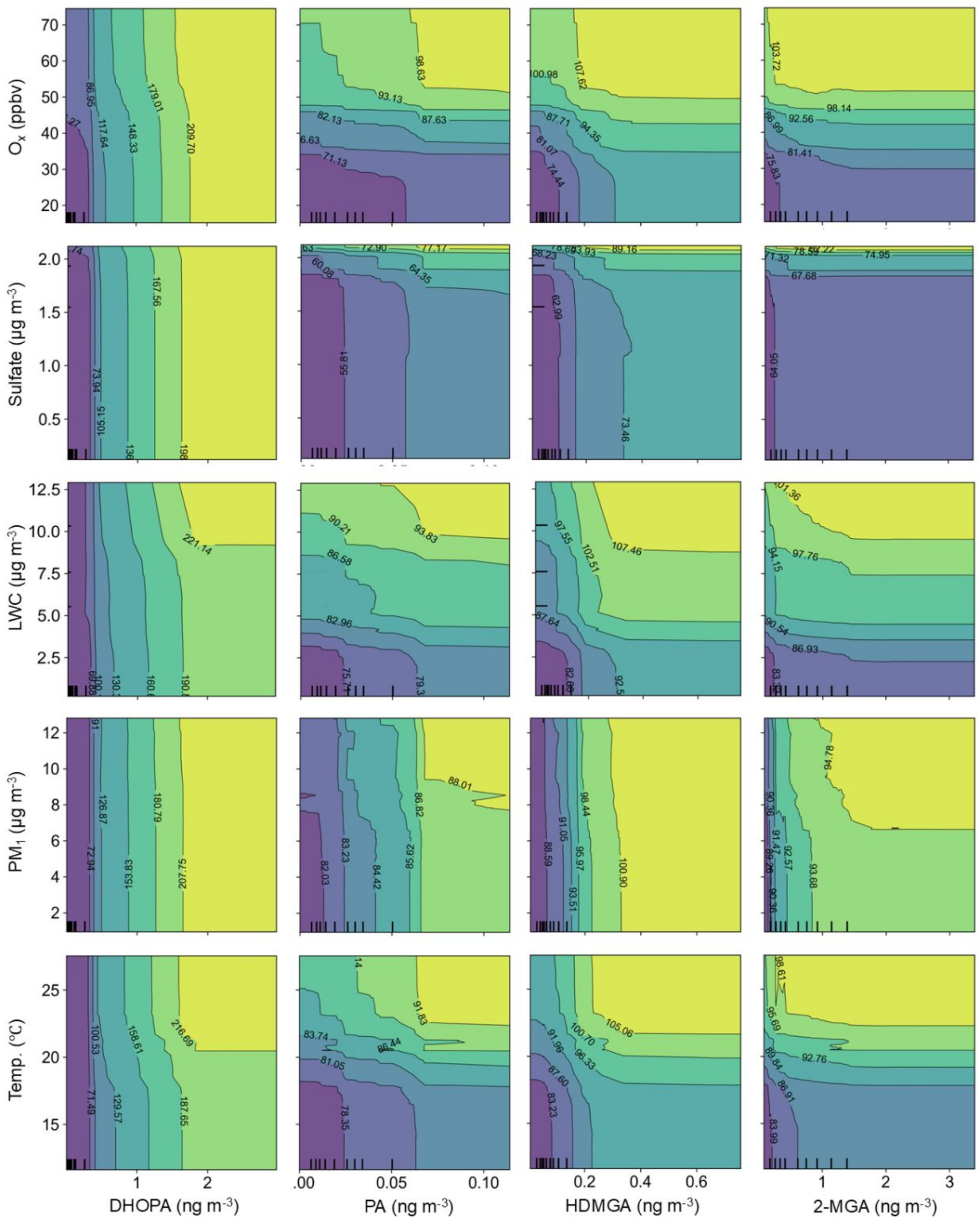

Figure S10. Responses of malic acid concentrations to the pairs of potential precursors and factors regulating chemical reactions.

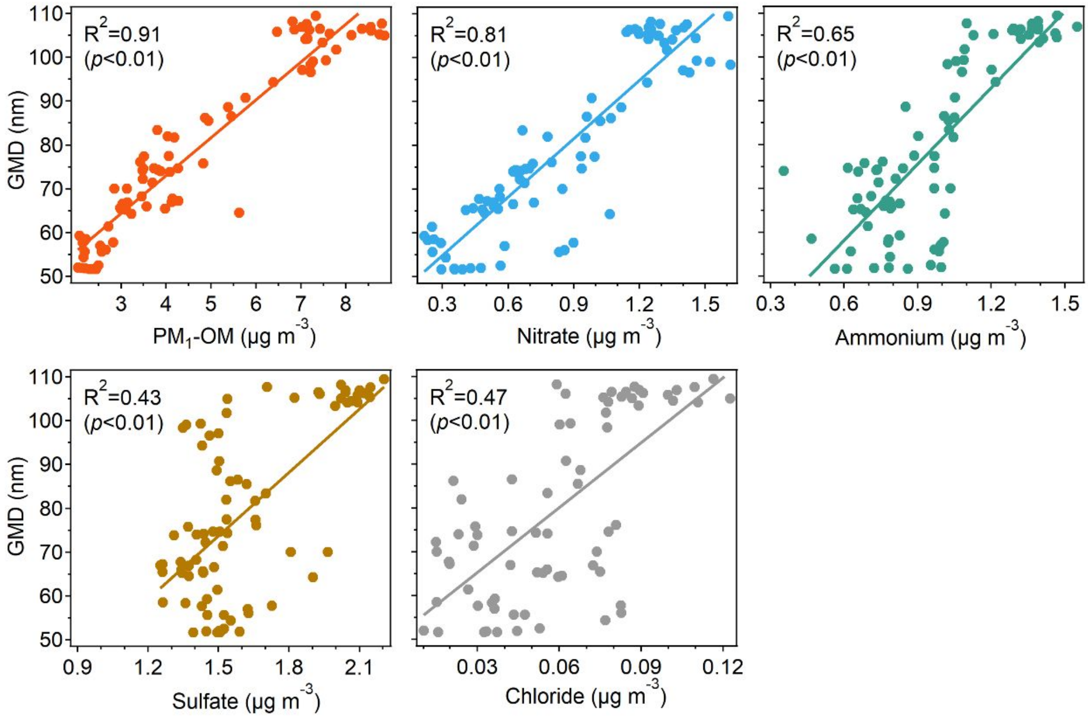

Figure S11. Correlations of GMD with non-refractory PM_1 components in the marine air case.

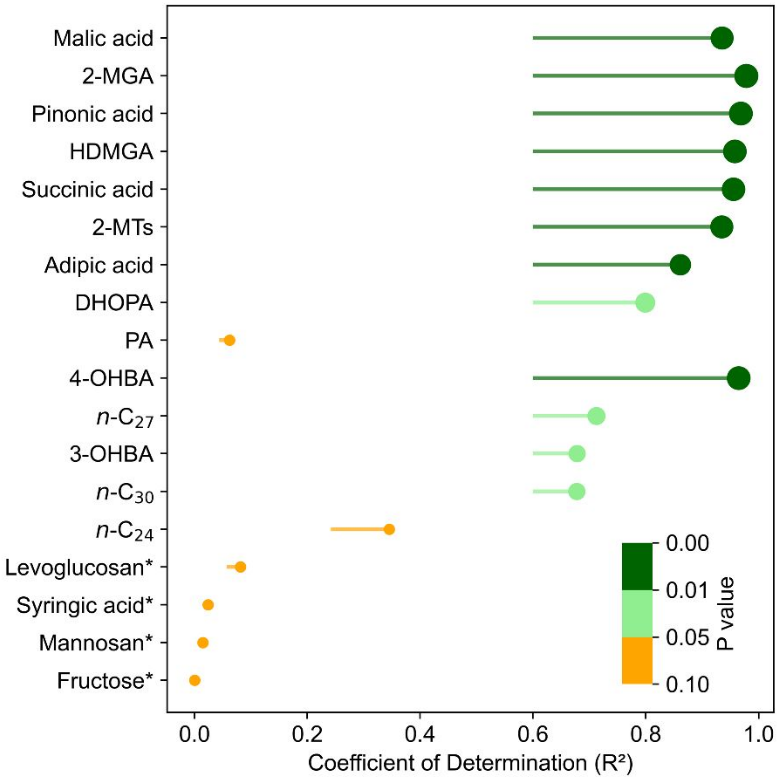

Figure S12. Coefficient of determination (R^2) for the correlation between GMD and OA markers measured by the TAG in the marine air case. Asterisks indicate opposite relationships.

Table S1. Details of the identification and quantification metrics for selected organic aerosol molecular markers. In bold are the OHDCA species we focus on in this study.

Compound	Base ion (m/z)	Retention time (s)	Internal standard	Surrogate standard	R^2 of Calibration Curve	Average concentration (ng m^{-3})
**Malic acid (MA)**	**233**	**677.3**	**Pentaerythritol-13C**	*	**0.7578**	**18.6 ± 7.9**
**Citramalic acid (CMA)**	**247**	**668.4**	**Pentaerythritol-13C**	*	**0.8637**	**1.3 ± 0.6**
**2-hydroxyglutaric acid (2-HGA)**	**349**	**722.9**	**Pentaerythritol-13C**	**Citramalic acid**	**0.8637**	**3.7 ± 1.6**
**Tartaric acid (TA)**	**292**	**732.1**	**Pentaerythritol-13C**	*	**0.7215**	**160.2 ± 53.4**
2-methylglyceric acid (2-MGA)	219	569.8	PentaerythrItol-13C	Citramalic acid	0.8637	0.7 ± 0.1
2-methylerythritol (2-MT1)	219	702.0	Pentaerythritol-13C	Citramalic acid	0.8637	2.7 ± 1.1
2-methylthreitol (2-MT2)	219	714.9	Pentaerythritol-13C	Citramalic acid	0.8637	6.7 ± 2.9
Pinic acid (PA)	171	770.8	1-Dodecand25-ol	Pinonic acid	0.9297	0.023 ± 0.004
3-hydroxy-4,4-dimethylglutaric acid (HDMGA)	377	744.4	Pentaerythritol-13C	Citramalic acid	0.8637	0.09 ± 0.02
2,3-dihydroxy-4-oxopentanoic acid (DHOPA)	321	693.3	Pentaerythritol-13C	Citramalic acid	0.8637	0.17 ± 0.09
Levoglucosan	333	790.2	Pentaerythritol-13C	*	0.9419	89.7 ± 25.8
Mannosan	333	779.4	Pentaerythritol-13C	*	0.9365	2.4 ± 0.8
4-hydroxybenzoic acid (4-OHBA)	267	744.8	1-Dodecan-d25-ol	*	0.9419	0.84 ± 0.08
Triacontane (n-C_{30})	57	1233.0	Triacontane-d62	Triacontane-d62	0.9731	0.14 ± 0.00

* Authentic standards were used for quantification.

Table S2. Instruments and working principles for real-time measurement of trace gases.

Measurement species	Instrument	Model	Working principle
CO	Teledyne Advanced Pollution Instrumentation (API) Gas Filter Correlation CO Analyzer	T300U	Infrared absorption
NO-NO_2-NO_x	Teledyne Advanced Pollution Instrumentation (API) NO/NO_x Analyzer	Model 200E	Chemiluminescence
O_3	Teledyne Advanced Pollution Instrumentation (API) O_3 Analyzer	T400	Ultraviolet absorption

Table S3. Independent variables as input of the RF model and their implications.

Variables	Implications
DHOPA	Aromatics and their oxidation intermediates/products as OHDCA precursors and/or similar formation pathways between OHDCA and DHOPA
PA	Monoterpenes and their oxidation intermediates/products (earlier generation oxidation products) as OHDCA precursors and/or similar formation pathways between OHDCA and PA
HDMGA	Monoterpenes and their oxidation intermediates/products (later generation oxidation products) as OHDCA precursors and/or similar formation pathways between OHDCA and HDMGA
2-MGA	Isoprene and its oxidation intermediates/products (high-NO_x) as OHDCA precursors and/or similar formation pathways between OHDCA and 2-MGA
4-OHBA	Biomass burning as a source of OHDCA
n-C_{30}	Alkanes as potential precursors
LWC	Aqueous processes
Sulfate	Aqueous processes
O_x	Photochemical processes
PM_1	Particle surface area
Temp.	Temperature

Table S4. Mean values of OA markers, trace gases and meteorological parameters in different types of air masses.

Species	Marine air	Coastal air	Continental air
CO (ppbv)	247 ± 20	223 ± 22	221 ± 9
O_3 (ppbv)	38.6 ± 5.8	37.3 ± 3.3	39.9 ± 2.5

NO (ppbv)	0.9 ± 0.6	0.2 ± 0.1	0.7 ± 0.2
NO_2 (ppbv)	3.6 ± 1.2	1.0 ± 0.6	3.9 ± 1.2
NO_x (ppbv)	4.5 ± 1.4	1.2 ± 0.6	4.6 ± 1.2
O_x (ppbv)	42.2 ± 6.0	38.3 ± 3.8	43.7 ± 2.7
Temp (°C)	22.9 ± 1.6	22.2 ± 1.8	16.8 ± 0.6
RH (%)	80.9 ± 5.4	84.1 ± 5.0	51.7 ± 3.2
Solar radiation (W m^{-2})	163.7 ± 130.4	200.1 ± 199.5	184.3 ± 55.3
WS (m s^{-1})	0.4 ± 0.2	0.8 ± 0.2	0.8 ± 0.1
LWC (µg m^{-3})	5.9 ± 2.0	5.1 ± 1.4	1.4 ± 0.4
PM_1-OM (µg m^{-3})	3.8 ± 1.3	1.8 ± 0.4	3.3 ± 0.4
Nitrate (µg m^{-3})	0.7 ± 0.3	0.1 ± 0.0	0.5 ± 0.1
Sulfate (µg m^{-3})	1.7 ± 0.1	1.9 ± 0.0	1.2 ± 0.1
Ammonium (µg m^{-3})	0.9 ± 0.1	0.9 ± 0.0	0.6 ± 0.1
Chloride (µg m^{-3})	0.049 ± 0.014	0.022 ± 0.005	0.024 ± 0.004
OHDCA (ng m^{-3})	554.2 ± 374.7	535.4 ± 189.0	89.5 ± 14.1
MA (ng m^{-3})	74.4 ± 51.9	45.3 ± 14.3	7.5 ± 1.4
CMA (ng m^{-3})	5.5 ± 3.9	3.8 ± 1.5	0.4 ± 0.1
2-HGA (ng m^{-3})	14.7 ± 10.5	8.0 ± 2.9	1.6 ± 0.3
TA (ng m^{-3})	459.6 ± 309.8	478.4 ± 170.7	79.4 ± 12.6
2-MGA (ng m^{-3})	0.98 ± 0.59	0.30 ± 0.07	0.67 ± 0.10
2-MTs (ng m^{-3})	47.9 ± 20.8	15.4 ± 9.6	2.9 ± 0.3
PA (ng m^{-3})	0.035 ± 0.016	0.055 ± 0.023	0.018 ± 0.003
HDMGA (ng m^{-3})	0.24 ± 0.16	0.09 ± 0.04	0.07 ± 0.01
DHOPA (ng m^{-3})	0.68 ± 0.59	0.21 ± 0.12	0.08 ± 0.02
Levoglucosan (ng m^{-3})	130.0 ± 46.2	19.4 ± 8.8	89.8 ±30.9
Mannosan (ng m^{-3})	3.2 ± 0.9	0.9 ± 0.2	2.4 ± 0.9
4-OHBA (ng m^{-3})	0.94 ± 0.20	0.68 ± 0.18	0.84 ± 0.09
2-MTs/2-MGA ratio	48	50	4
